# Electroacupuncture Modulates Spinal BDNF/TrκB Signaling Pathway and Ameliorates the Sensitization of Dorsal Horn WDR Neurons in Spared Nerve Injury Rats

**DOI:** 10.3390/ijms21186524

**Published:** 2020-09-07

**Authors:** Meng Xue, Ya-Lan Sun, Yang-Yang Xia, Zhi-Hua Huang, Cheng Huang, Guo-Gang Xing

**Affiliations:** 1 Department of Physiology, Basic Medical College, Gannan Medical University, Ganzhou 341000, China; mengxue787@gmail.com (M.X.); yalan.sun558@gmail.com (Y.-L.S.); gnyxyxyy@gmu.edu.cn (Y.-Y.X.); zh.huang@gmu.edu.cn (Z.-H.H.); 2 Pain Medicine Research Institute, Gannan Medical University, Ganzhou 341000, China; 3 Neuroscience Research Institute, Peking University, Beijing 100191, China

**Keywords:** electroacupuncture, BDNF/TrκB signaling pathway, neuropathic pain, WDR neuron, spinal cord

## Abstract

Neuropathic pain is more complex and severely affects the quality of patients’ life. However, the therapeutic strategy for neuropathic pain in the clinic is still limited. Previously we have reported that electroacupuncture (EA) has an attenuating effect on neuropathic pain induced by spared nerve injury (SNI), but its potential mechanisms remain to be further elucidated. In this study, we designed to determine whether BDNF/TrκB signaling cascade in the spinal cord is involved in the inhibitory effect of 2 Hz EA on neuropathic pain in SNI rats. The paw withdrawal threshold (PWT) of rats was used to detect SNI-induced mechanical hypersensitivity. The expression of BDNF/TrκB cascade in the spinal cord was evaluated by qRT-PCR and Western blot assay. The C-fiber-evoked discharges of wide dynamic range (WDR) neurons in spinal dorsal horn were applied to indicate the noxious response of WDR neurons. The results showed that 2 Hz EA significantly down-regulated the levels of BDNF and TrκB mRNA and protein expression in the spinal cord of SNI rats, along with ameliorating mechanical hypersensitivity. In addition, intrathecal injection of 100 ng BDNF, not only inhibited the analgesic effect of 2 Hz EA on pain hypersensitivity, but also reversed the decrease of BDNF and TrκB expression induced by 2 Hz EA. Moreover, 2 Hz EA obviously reduced the increase of C-fiber-evoked discharges of dorsal horn WDR neurons by SNI, but exogenous BDNF (100 ng) effectively reversed the inhibitory effect of 2 Hz EA on SNI rats, resulting in a remarkable improvement of excitability of dorsal horn WDR neurons in SNI rats. Taken together, these data suggested that 2 Hz EA alleviates mechanical hypersensitivity by blocking the spinal BDNF/TrκB signaling pathway-mediated central sensitization in SNI rats. Therefore, targeting BDNF/TrκB cascade in the spinal cord may be a potential mechanism of EA against neuropathic pain.

## 1. Introduction

Neuropathic pain is a type of chronic pain syndrome induced by peripheral or central nervous system injuries, lesions, and dysfunction [1]. In clinical practice, neuropathic pain is great correlation with hyperalgesia, allodynia, and spontaneous pain [2], which causes a great burden to patients’ lives mainly by impairing sleep quality and producing adverse emotions, depression, and cognitive disorders [3]. Since the development and maintenance of neuropathic pain is extremely complicated, coupled with its long period of clinical therapy and unsatisfactory effect [4], it is of great significance to further clarify the formation process of neuropathic pain and its pathogenesis.

It is evident that brain-derived neurotrophic factor (BDNF), an important member of the neurotrophic factor family, is involved in maintaining synaptic plasticity and central sensitization, which plays a crucial role in the induction of chronic pain [5,6,7,8]. BDNF has also been reported to be widely distributed in the pain-related pathways and participates in pain signaling transmission and modulation [9]. It was found that BDNF expression was significantly up-regulated in the spinal cord and dorsal root ganglia (DRG) of rats following peripheral nerve injury [6,7,10]. Further experiments demonstrated that BDNF exerts its biological effect mainly through binding to its high-affinity tropomyosin related kinase B (TrκB) receptor and subsequently activating the downstream signaling cascade [11,12]. Additionally, the spinal BDNF/TrκB signaling pathway is implicated in the induction of neuropathic pain following SNL-evoked neuropathic pain [9,13]. Central sensitization is identified to be an important factor in the pathogenesis of neuropathic pain [14,15], which promotes the excitability of nociceptive signaling cascade in pain-related neurons [15]. It is clear the effects of spinal BDNF on the C-fiber responses of dorsal horn wide dynamic range (WDR) neurons, which are closely associated with nociceptive transmission and central sensitization [16,17]. Administration of BDNF produced hyperexcitability of spinal dorsal horn WDR neurons as determined by detecting C-fiber-evoked discharges, the increased BDNF in the spinal cord leads to the development of hyperexcitability of dorsal horn WDR neurons as well as pain hypersensitivity after spinal nerve injury [9]. Overall, these data suggested that BDNF/TrκB signaling cascade-mediated central sensitization plays a critical role in neuropathic pain development.

Owing to the complicated pathophysiology of neuropathic pain, its clinical management is extremely challenging. With deepen research on the pathogenesis of neuropathic pain, its therapeutic approaches mainly focus on signaling cascades and molecular targets that induce pain hypersensitivity. Currently, the available treatment against neuropathic pain may mostly rely on drugs. According to the latest recommendation of International Association for Study of Pain in treating neuropathic pain, the commonly used first-line drugs are mainly SNRI (duloxetine and venlafaxine), cyclic antidepressants as well as gabapentin and pregabalin [18]. However, these drugs have many side effects, such as neurocognition, falls, and weight gain [18]. In addition, the management of neuropathic pain lasts for long-term, and most patients have not enough to relieve pain [19,20]. Therefore, it is particularly urgent and important to investigate more effective and novel therapeutic options for neuropathic pain.

It is well known that electroacupuncture (EA), developed from traditional acupuncture, has been widely used in China and some Asian countries for clinical practice due to its considerable fewer side effects [21,22]. Evidence showed that EA stimulation has an effectiveness on neuropathic pain [23,24]. For example, neurotransmitters such as neurotrophic and cytokine factors are involved in EA producing an attenuation of neuropathic pain [25,26]. Stimulation with 2 Hz EA alleviates SNL-induced neuropathic pain by promoting the occurrence of long-term depression (LTD) in the spinal dorsal horn [27]. Our previous studies have demonstrated that 2 Hz EA successfully ameliorated mechanical hypersensitivity through blockade of microglial activation and pro-inflammatory cytokine IL-1β production in the spinal cord of SNI rats [23,28,29], but the underlying mechanisms remain largely unknown. Based on the fact that neuroinflammatory response is regarded as a crucial role in the pathogenesis of neuropathic pain, including the alterations of pain-related signaling molecules [30,31], BDNF is known to be an endogenous neuromodulator in the nociceptive pathway and exerts a critical action via its interactions with TrκB receptor [8], and the BDNF/TrκB signaling pathway plays an important role in central sensitization and neuropathic pain development. Accordingly, we hypothesized that 2 Hz EA stimulation may exhibit its analgesic effect by inhibiting spinal BDNF/TrκB signaling pathway-mediated central sensitization in SNI-induced neuropathic pain in rats.

Therefore, this study aimed to investigate whether the analgesic mechanisms of 2 Hz EA is associated with suppression of the hyperexcitability of dorsal horn WDR neurons mediated by the BDNF/TrκB signaling pathway in SNI rats. Following intrathecal administration of exogenous BDNF to SNI rats, we further defined the effect of 2 Hz EA stimulation on SNI rats. This experiment will gain insight into the possible role of BDNF/TrκB signaling pathway in 2 Hz EA-mediated alleviation of neuropathic pain, and BDNF/TrκB maybe served as a promising target for EA management of neuropathic pain.

## 2. Results

### 2.1. BDNF Reversed the Inhibitory Effect of 2 Hz EA on Mechanical Hypersensitivity in SNI Rats

**Figure 1:**
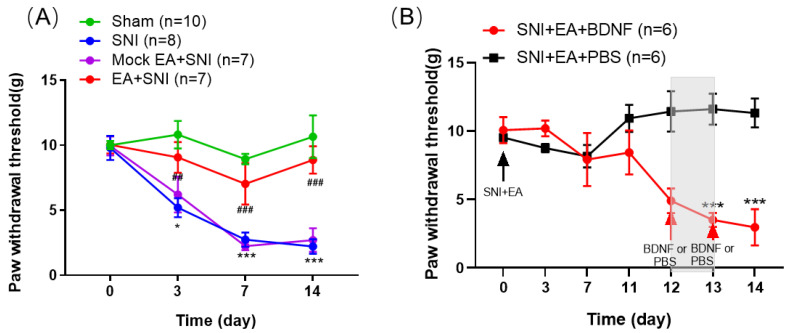
2 Hz electroacupuncture (EA) treatment relieved spared nerve injury (SNI)-induced mechanical hypersensitivity in rats. (**A**) Rats were treated with 2 Hz EA, starting from day 1 post-SNI surgery, once every other day lasting for 14 days (*n* = 7–10). The PWT was measured on days 0, 3, 7, and 14 post-SNI operation. Sham represents the sham group; SNI represents rats treated with the spared nerve injury-elicited neuropathic pain; SNI+EA represents rats receiving spared nerve injury and EA treatment. Mock EA + SNI represents rats receiving spared nerve injury and EA only stimulates acupoints, no electrical stimulation. (**B**) Following 2 Hz EA treatment of SNI rats, intrathecally injected 100 ng BDNF or PBS to SNI rats on day 12 post-surgery. BDNF + EA + SNI and PBS + EA + SNI represents rats receiving intrathecal administration of 100 ng BDNF or PBS (*n* = 6 per group). * *p* < 0.05, *** *p* < 0.001, compared with the sham group. ^##^ *p* < 0.01, ^###^ *p* < 0.001, compared with the SNI group. All data were expressed as mean ± SEM, two-way repeated-measures ANOVA was followed by Bonferroni’s post-hoc test.

In this experiment, we explored the potential efficacy of 2 Hz EA in SNI-induced neuropathic pain in rats. The results are shown in Figure 1A. Compared with the sham group, the PWT in the SNI group was significantly reduced (*p* < 0.05, *p* < 0.001). Compared with the SNI group, after 2 Hz EA stimulation was given to SNI rats, The PWT of rats in the SNI+EA group was significantly increased (*p* < 0.01, *p* < 0.001), while the PWT of rats in the SNI + Mock EA group has no significant difference compared with the SNI group. These findings revealed that 2 Hz EA alleviates the mechanical hypersensitivity in SNI-induced neuropathic pain rats. In order to further examine the effect of exogenous BDNF in the inhibitory effect of 2 Hz EA on SNI-evoked mechanical hypersensitivity, intrathecally injected 100 ng BDNF or PBS on SNI rats starting from day 12 of 2 Hz EA treatment. The results are shown in Figure 1B. Compared with intrathecal administration of PBS, the PWT of rats given BDNF is significantly decreased (*p* < 0.001). The data indicated that BDNF can reverse the inhibitory effects of 2 Hz EA on mechanical hypersensitivity in rats following SNI.

### 2.2. 2 Hz EA Induced the Down-Regulation of BDNF mRNA and Protein Levels in the Spinal Cord of SNI Rats

**Figure 2:**
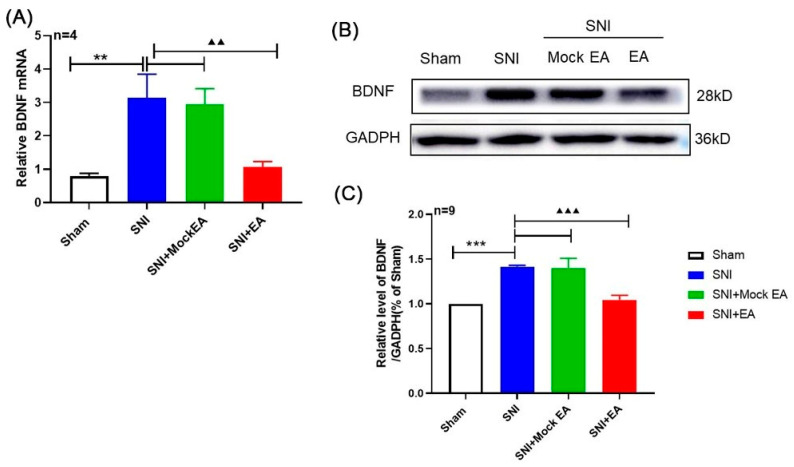
2 Hz EA treatment reversed the SNI-induced up-regulation of brain-derived neurotrophic factor (BDNF) in the spinal cord. (**A**) Relative levels of BDNF mRNA expression (*n* = 4 per group). (**B**) Western blotted band of BDNF. (**C**) The relative level of BDNF protein expression (*n* = 9 per group). SNI increased BDNF protein expression in the spinal cord, which was attenuated by 2 Hz EA treatment. Samples were collected from the spinal cord of L4–L6 segments on day 14. ** *p* < 0.01, *** *p* < 0.001, compared with the Sham group. ^▲▲^ *p* < 0.01, ^▲▲▲^ *p* < 0.001, compared with the SNI group. All data were expressed as mean ± SEM, one-way ANOVA was followed by the Newman–Keuls post-hoc test.

In order to investigate the effect of 2 Hz EA on the spinal BDNF in SNI rats, qRT-PCR and Western blot assay were used to determine the mRNA and protein levels of BDNF in the spinal cord of SNI rats on day 14 after 2 Hz EA treatment. As shown in Figure 2A, compared with the sham group, the mRNA expression of BDNF in the spinal cord of SNI rats was significantly increased on day 14 after SNI operation (*p* < 0.01). After 2 Hz EA treatment of SNI rats, the level of BDNF mRNA in the spinal cord of rats in the SNI + EA group was obviously down-regulated (*p* < 0.01). However, the mRNA expression of BDNF in the spinal cord of rats from the SNI+Mock EA group was not different from that of SNI group. Similarly, as shown in Figure 2B,C, compared with the sham group, the protein expression of BDNF in the spinal cord of the SNI group rats was markedly up-regulated on the 14th day following SNI (*p* < 0.001). After 2 Hz EA treatment, the protein level of BDNF in the spinal cord of rats in the SNI+EA group was markedly reduced (*p* < 0.001). However, the protein expression of BDNF in the spinal cord of rats from the SNI + Mock EA group was not different from that of SNI group. These results demonstrated that 2 Hz EA can down-regulate the expression levels of BDNF mRNA and protein in the spinal cord of SNI rats.

### 2.3. Intrathecal Administration of Exogenous BDNF Reversed the 2 Hz EA-Induced Down-Regulation of BDNF mRNA and Protein Levels in the Spinal Cord of SNI Rats

**Figure 3:**
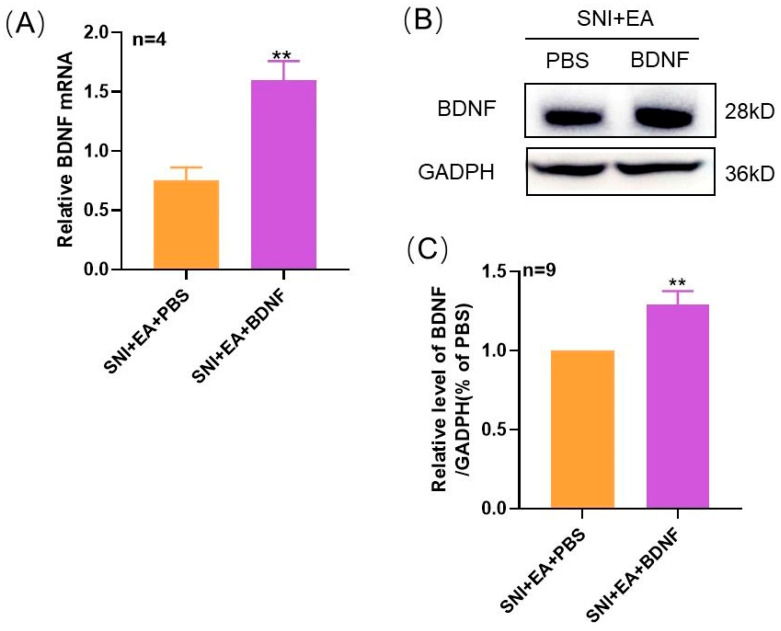
Effect of exogenous BDNF on spinal BDNF mRNA and protein expression induced by 2 Hz EA in SNI rats. (**A**) Relative levels of BDNF mRNA expression (*n* = 4 per group). (**B**) Western blotted band of BDNF. (**C**) The relative level of BDNF protein expression (*n* = 9 per group). Intrathecal administration of 100 ng BDNF or PBS restored the decreased expression of BDNF in the SNI+EA group. Samples were collected from the spinal cord of L4-L6 segments on day 14. ** *p* < 0.01, compared with the SNI + EA + PBS group. All data were expressed as mean ± SEM, one-way ANOVA was followed by the Newman–Keuls post-hoc test.

In order to further verify whether the effect of 2 Hz EA on SNI-induced neuropathic pain through BDNF signaling, on the 12th day of 2 Hz EA treatment to SNI rats, we intrathecally injected 100 ng BDNF or PBS for 2 days, and then conducted the spinal cord of SNI rats on the 14th day to detect the levels of BDNF mRNA and protein expression. The results are shown in Figure 3A. Compared with intrathecal injection of PBS, the mRNA expression of BDNF in the spinal cord of SNI rats following exogenous BDNF was significantly up-regulated (*p* < 0.01). Similarly, as shown in Figure 3B,C, compared with the SNI + EA + PBS group, the expression level of BDNF in the spinal cord of rats in the SNI+EA+BDNF group was obviously increased (*p* < 0.01). These results showed that exogenous BDNF can reverse the expression of endogenous BDNF induced by 2 Hz EA in SNI rats.

### 2.4. 2 Hz EA Induced the Reduction of TrκB mRNA and Protein Levels in Spinal Cord of SNI Rats

**Figure 4:**
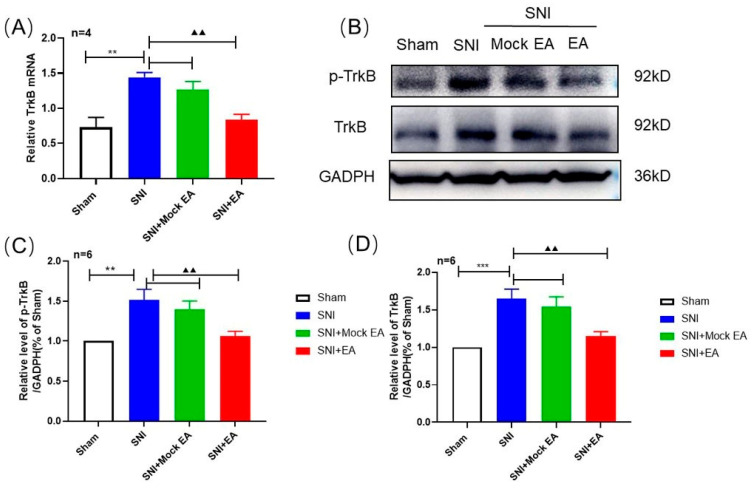
2 Hz EA treatment attenuated the SNI-induced up-regulation of p-TrκB and TrκB expression in the spinal cord. (**A**) The relative level of TrκB mRNA expression (*n* = 4 per group). (**B**) Western blotted band of p-TrκB and TrκB. (**C**) The relative level of p-TrκB protein expression (n = 6 per group). (**D**) The relative level of TrκB protein expression (*n* = 6 per group). SNI increased the p-TrκB and TrκB protein expression in the spinal cord, which was inhibited by 2 Hz EA treatment. Samples were collected from spinal cord of L4-L6 segments of rats on day 14. ** *p* < 0.01, ***p < 0.001, compared with the sham group. ^▲▲^ *p* < 0.01, compared with the SNI group. All data were expressed as mean ± SEM, one-way ANOVA was followed by Newman–Keuls post-hoc test.

To observe the effect of 2 Hz EA on spinal TrκB in SNI rats, on the 14th day after SNI, qRT-PCR, and Western blot were applied to detect the mRNA and protein expression of spinal TrκB by 2 Hz EA in SNI rats. These results are shown in Figure 4A. Compared with the sham group, the mRNA expression of TrκB in the spinal cord of rats in the SNI group was markedly enhanced (*p* < 0.01). Compared with the SNI group, the mRNA expression of TrκB in the spinal cord of SNI+EA group rats was significantly down-regulated (*p* < 0.01), but compared with the SNI group, the mRNA expression level of TrκB in the spinal cord of rats from the SNI + Mock EA group did not significantly change. Similarly, as shown in Figure 4B–D, compared with the sham group, the expression levels of p-TrκB and TrκB in the spinal cord of the SNI group rats were obviously up-regulated (*p* < 0.01, *p* < 0.001), compared with the SNI group, the protein expression of p-TrκB and TrκB in the spinal cord of SNI + EA group rats was significantly reduced (*p* < 0.01); however, compared with the SNI group, the protein expression levels of p-TrκB and TrκB in the spinal cord of SNI + Mock EA group rats were not obvious change. These findings indicated that 2 Hz EA significantly inhibited the TrκB mRNA and protein expression in the spinal cord of SNI rats.

### 2.5. Intrathecal Injection of Exogenous BDNF Reversed the 2 Hz EA-Induced Down-Regulation of TrκB mRNA and Protein Levels in the Spinal Cord of SNI Rats

**Figure 5:**
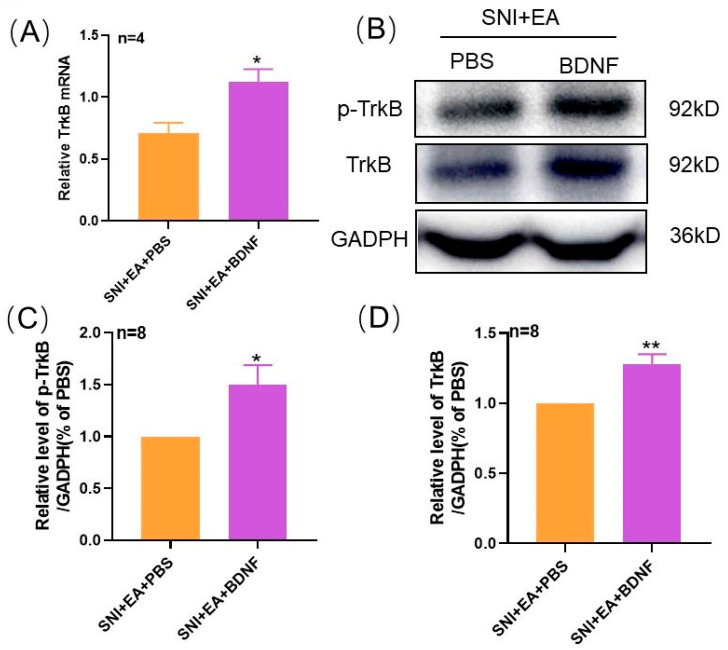
Effect of exogenous BDNF or PBS on spinal p-TrκB and TrκB protein expression induced by 2 Hz EA in SNI rats. (**A**) Relative levels of TrκB mRNA expression (*n* = 4 per group). (**B**) Western blotted band of p-TrκB and TrκB. (**C**) The relative level of p-TrκB protein expression (*n* = 8 per group). (**D**) The relative level of TrκB protein expression (*n* = 8 per group). Intrathecal administration of 100 ng BDNF or PBS restored the decreased expression of p-TrκB and TrκB in the SNI + EA group. Samples were collected from the spinal cord of L4–L6 segments on day 14. * *p* < 0.05, ** *p* < 0.01, compared with the SNI + EA + PBS group. All data were expressed as mean ± SEM, one-way ANOVA was followed by the Newman–Keuls post-hoc test.

In order to further explore the effect of exogenous BDNF on TrκB expression in the spinal cord of SNI rats by 2 Hz EA, after 2 Hz EA treatment of SNI rats, we intrathecally injected with BDNF or PBS to SNI rats from day 12. On the 14th day, the spinal cord of SNI rats was collected to detect the levels of TrκB mRNA and protein expression. The results are shown in Figure 5A. Compared with PBS, after intrathecal injection of exogenous 100 ng BDNF, the level of TrκB mRNA in the spinal cord of SNI rats was significantly increased (*p* < 0.05). Similarly, as shown in Figure 5B–D, compared with the SNI + EA + PBS group, after intrathecal injection of exogenous BDNF, the protein expression levels of p-TrκB and TrκB in the spinal cord of rats in the SNI + EA + BDNF group were markedly increased (*p* < 0.05, *p* < 0.01). These results showed that exogenous BDNF effectively reversed the inhibitory effect of 2 Hz EA on TrκB expression in the spinal cord of SNI rats.

### 2.6. 2 Hz EA Decreased the C-Fiber-Evoked Discharges of Dorsal Horn WDR Neurons in SNI Rats

**Figure 6:**
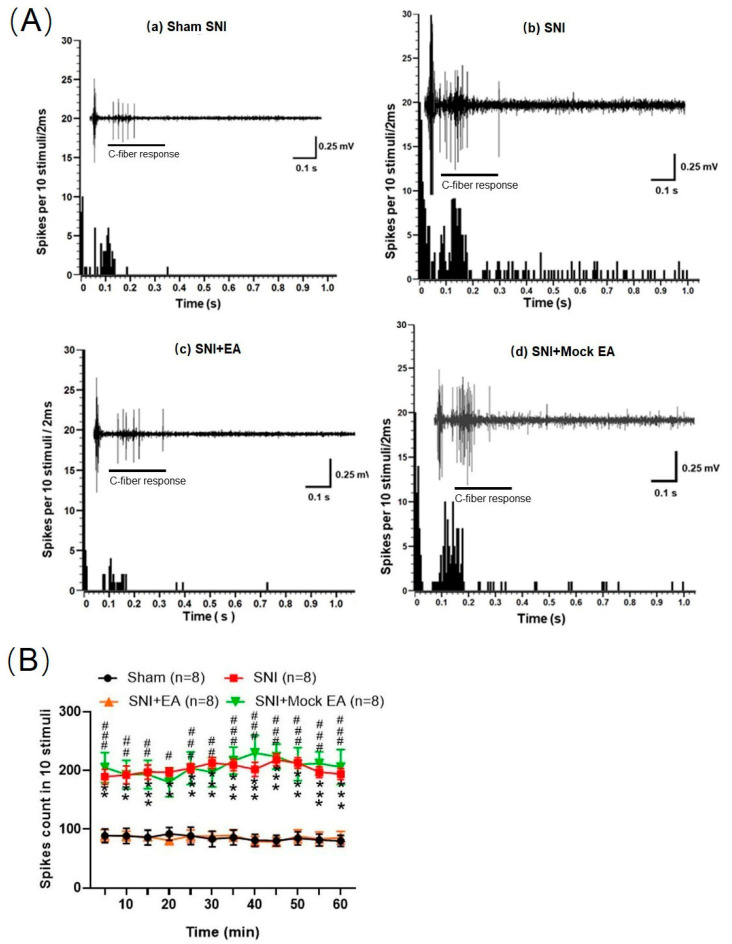
Effects of 2 Hz EA treatment on the C-fiber responses of dorsal horn wide dynamic range (WDR) neurons in SNI rats. (**A**) The original recording on the C-fiber-evoked discharges of a WDR neuron before and after 2 Hz EA treatment in SNI rats. a: Representative C-fiber responses of dorsal horn WDR neurons in the sham group; b: SNI, spared nerve injury surgery; c: SNI+EA, 2 Hz EA treatment; d. Mock EA+SNI, EA only stimulates acupoints, no electrical stimulation. (**B**) The total spikes count of the electrically evoked C-fiber responses in 10 stimuli. ** *p* < 0.01, *** *p* < 0.001, compared to the sham group. ^#^ *p* < 0.05, ^##^ *p* < 0.01, ^###^ *p* < 0.001, compared to the SNI group, respectively. Two-way ANOVA followed by Bonferroni’s post-hoc test, *n* = 8 per group.

To investigate the effect of 2 Hz EA on the excitability of WDR neurons in the spinal dorsal horn of SNI rats. Extracellular electrophysiological recording was performed to determine the alteration of C-fiber-evoked discharges of dorsal horn WDR neurons in SNI rats. The results are shown in Figure 6. Compared with the sham group, the C-fiber-evoked discharges of dorsal horn WDR neurons in SNI rats were significantly enhanced. Within 0–60 min, each string of stimulation was induced 10 times, the total number of discharges was significantly different from the sham group (*p* < 0.01, *p* < 0.001). Compared with the SNI group, the C-fiber-evoked discharges of dorsal horn WDR neurons in the SNI+EA group rats were significantly reduced. Compared with the SNI group, the sum of the numbers of C-fiber-evoked discharges was significantly different (*p* < 0.05, *p* < 0.01, *p* < 0.001). Compared with the SNI group, the dorsal horn WDR neurons in rats from the SNI+Mock EA group has no significant difference in C-fiber-evoked discharges. These results revealed that the excitability of WDR neurons in the spinal dorsal horn of SNI rats was obviously enhanced, while 2 Hz EA can suppress the excitability of dorsal horn WDR neurons in SNI rats.

### 2.7. Intrathecal Injection of BDNF Reversed the 2 Hz EA-Induced Reduction of C-Fiber-Evoked Discharges of Dorsal Horn WDR Neurons in SNI Rats

**Figure 7:**
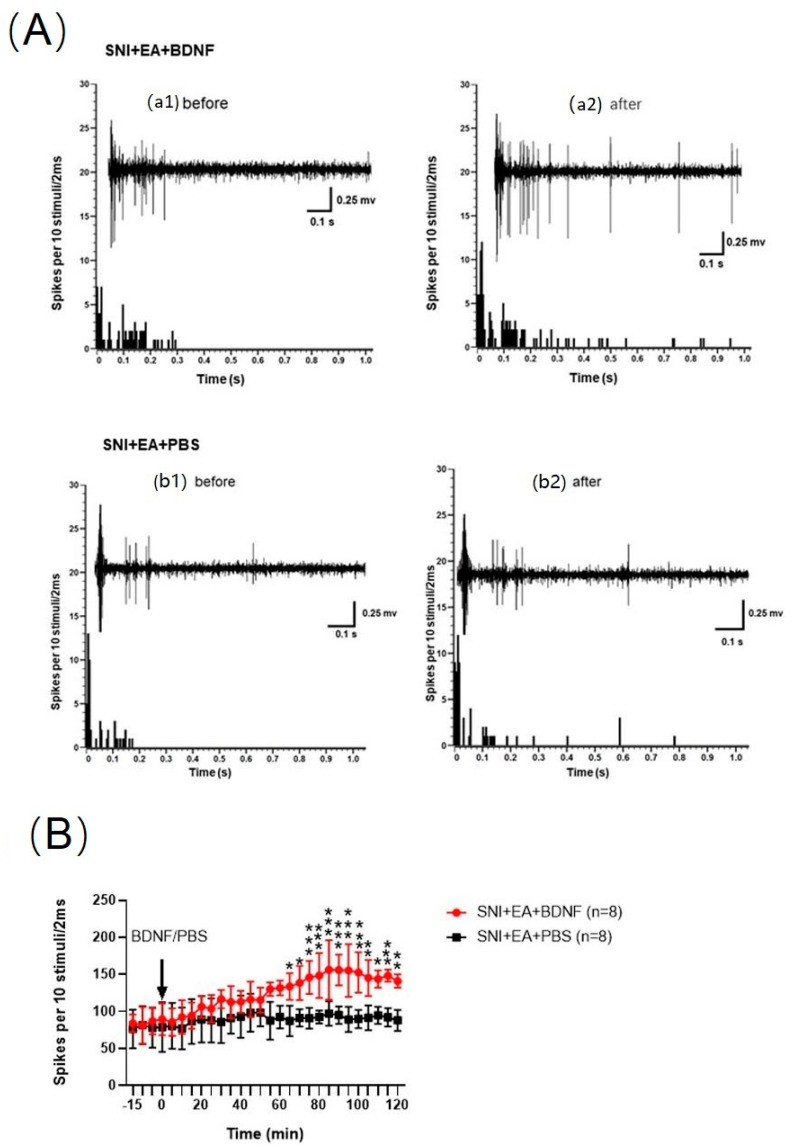
Effects of spinal administration of BDNF on the C-fiber responses of dorsal horn WDR neurons induced by 2 Hz EA treatment. (**A**) The alterations of C-fiber-evoked discharges of a WDR neuron induced by 2 Hz EA before and after administration of PBS or 100 ng BDNF. a1: 15 min after or before PBS administration (baseline); a2: 120 min after PBS administration; b1: 15 min after or before BDNF administration (baseline); b2: 120 min after BDNF administration. (**B**) The total spikes count of the electrically evoked C-fiber responses in 10 stimuli. * *p* < 0.05, ** *p* < 0.01, *** *p* < 0.001, compared to the SNI + EA + PBS group, respectively, two-way ANOVA followed by Bonferroni’s post-hoc test, *n* = 8 per group.

To further elucidate the potential role of BDNF/TrκB signaling pathway in 2 Hz EA treatment of neuropathic pain. The results are shown in Figure 7. Following given exogenous 100 ng BDNF on the spinal dorsal horn of SNI rats, we found that BDNF can significantly inhibit the effect of 2 Hz EA on the reduction of C-fiber-evoked discharges of dorsal horn WDR neurons (*p* < 0.05, *p* < 0.01, *p* < 0.001). However, after the same dose of PBS was administered to the spinal dorsal horn surface of SNI rats, the effect of 2 Hz EA on the reduction of C-fiber-evoked discharges in dorsal horn WDR neurons remained unchanged. These results indicated that exogenous BDNF given to the spinal dorsal horn surface of SNI rats successfully reversed the 2 Hz EA-induced reduction of C-fiber-evoked discharges of dorsal horn WDR neurons in SNI rats.

## 3. Discussion

Neuropathic pain is a common clinical chronic disease, and its etiology, pathogenesis, and initiation process are greatly complicated [32,33], which is mainly through peripheral and central nervous system damage or obstacles caused by a series of pathological symptoms [34]. Presently, neuropathic pain is still a serious health issue [32], and it is very urgent to search for more effective management of neuropathic pain.

It is well known that EA stimulation served as an subsidiary therapy has been widely used to relieve neuropathic pain with fewer side effects [22,29]. Experiments demonstrated that EA stimulation has an inhibitory effect on mechanical hypersensitivity in spinal nerve ligation (SNL) rats [9,35]. In the previous research, we also found that EA can regulate the activation of microglia in the spinal cord of SNI rats, thereby affecting the release of inflammatory cytokines and inhibiting pain-related signaling pathways [23,29,36]. Study showed that EA can promote neuroplasticity by increasing neurotrophic factors in the spinal cord [37]. In other studies, it was also found that 2 Hz EA caused LTD formation in the spinal cord of SNL rats [27]. Subsequently, the neurotrophic factors, inflammatory factors, neuroplasticity, and related signal pathways in rat DRG treated with EA are significantly regulated in neuropathic pain [28,38,39]. The analgesic effect induced by EA treatment is mainly dependent on EA stimulation-frequencies and -intervals. Previously reported that the inhibitory effect of 2 Hz EA stimulation on neuropathic pain is better than that in 100Hz EA [40]. Beyond that, alternating frequency stimulation (2/100Hz) EA has a better effect on inflammatory pain and internal organs [41,42]; however, 2 Hz EA has gained more recognition in the treatment of neuropathic pain [22]. Several results have further demonstrated that repeated or prolonged EA stimulation would lead to EA tolerance [43]. Therefore, we presently selected 2 Hz EA treatment of SNI-induced neuropathic pain rats with once every other day to avoid the tolerance to EA stimulation. This is consistent with the previous studies [28,29], in this research, following 2 Hz EA stimulation of the acupoints of Zusanli and Sanyinjiao in SNI rats, the PWT is significantly increased, indicating that 2 Hz EA indeed alleviated SNI-induced mechanical hypersensitivity. Additionally, with the extension of 2 Hz EA treatment, the analgesic effect was gradually improved, suggesting that 2 Hz EA stimulation has a cumulative relieving effect on SNI rats. Our present findings in the spinal cord are consistent with others. In general, 2 Hz EA can treat neuropathic pain in many ways, but the specific mechanism still needs to be explored.

It is known that BDNF is a member of the neurotrophic factor family, through its high affinity binding to TrκB receptor play a physiological role [44,45,46,47,48]. Experiments have shown that BDNF has two forms of proteolysis, which are divided into precursor BDNF (proBDNF) and mature BDNF (mBDNF). The precursor BDNF with a molecular weight of about 30kD can be cleaved into mature BDNF with a molecular weight of about 13kD under the action of plasmin. The precursor BDNF and mature BDNF have different physiological roles in the nervous system. Precursor BDNF can bind to the low-affinity receptor p75 to induce neuronal death, while the binding of mature BDNF to TrκB receptor can evoke neuronal survival [45,49]. In other reports, the presence of BDNF in the brain can promote nerve growth in late brain development, through NMDAR/Ca21-Dependent Signaling produce neuroprotection, and modifies synaptic plasticity [46,47,48]. Moreover, the activation of TrκB receptor also leads to the response of downstream signaling molecules, for example, Pi3k/Akt, MAPK and PLC-γ signaling pathways [48], thereby affecting cell proliferation, differentiation, apoptosis, and other physiological activities. This indicated that the BDNF/TrκB signaling pathway has an important role in the central nervous system. In addition, BDNF has a strong repairing effect on nerve plasticity and plays an important role in regulating pain [50]. How does the BDNF/TrκB signaling pathway participate in the modulation of neuropathic pain? The BDNF/TrκB signaling pathway in the spinal cord has been shown to play a critical role in central sensitization and neuropathic pain development, which attracts more attention in recent years [6,8,9]. For example, previous experiment has demonstrated that spinal BDNF interacting with its high-affinity TrκB receptor is an important process that ultimately leads to a pathological state [51]. Peripheral nerve injury induced a remarkable enhancement of BDNF expression and possessed a regulatory activity on the occurrence and development of neuropathic pain [52]. Accumulating evidence exhibited that under neuropathic pain states, BDNF expression is obviously increased in the spinal cord, and TrκB protein level is also markedly up-regulated within this process [13,53]. Nerve injury promoted the release of BDNF from microglia and binding to spinal TrκB receptor on dorsal horn neurons, decreasing the potassium chloride transporter KCC2, leading to the increase of intracellular Cl- and inhibition of GABAergic neurons, eventually participating in NMDA receptor-mediated central sensitization and inducing neuropathic pain [9,54]. Consistently, in this study, the levels of BDNF and TrκB mRNA and protein expression in the spinal cord of SNI rats were obviously increased, indicating that spinal BDNF/TrκB signaling pathway is likely to be involved in the formation of neuropathic pain induced by SNI. Meanwhile, other studies have reported that the expression of BDNF and TrκB in the spinal cord of CCI mice treated with EA stimulation was significantly reduced, together with the alleviation of pain hypersensitivity [26]. There is an interesting finding in the other studies, after removing the L1–L5 and L7–S2 spinal nerves on both sides, the level of BDNF in L6 DRG treated with EA was significantly increased [55], and found that BDNF in L3–L5 DRG is upregulated in SNI model [7]. This indicates that BDNF in different models of neuropathic pain may have different effects.

Similarly, we currently found that 2 Hz EA treatment of SNI rats resulted in a prominent down-regulation in BDNF and TrκB expression in both mRNA and protein in the spinal cord, suggesting that 2 Hz EA stimulation exerting the analgesic effect on SNI rats maybe through suppressing the BDNF/TrκB signaling pathway. Furthermore, the findings of intrathecal injection of exogenous BDNF to SNI rats showed that BDNF successfully reverses the inhibitory effect of 2 Hz EA on BDNF/TrκB signaling, leading to a notable improvement of BDNF and TrκB expression in both mRNA and protein in the spinal cord of SNI rats. Accordingly, this further confirmed that the therapeutic effect of 2 Hz EA on SNI-induced neuropathic pain is highly correlated with the BDNF/TrκB cascade, implying that 2 Hz EA stimulation induces the analgesic effect via blocking the BDNF/TrκB signaling pathway in the spinal cord of SNI rats.

In order to further explore whether the BDNF/TrκB signaling pathway inhibits the effect of 2 Hz EA on neuropathic pain. Thus, we conducted electrophysiological experiments on WDR neurons in SNI rats. Since the excitability of WDR neurons in the spinal dorsal horn is closely related to central sensitization [35]. There is evidence that WDR neurons are a class of non-specific nociceptive neurons that located in the deep dorsal horn, mainly in laminae IV and V of the spinal dorsal horn [35], and it receives signaling from Aβ and C fibers, which is highly associated with the pain signaling transmission and central sensitization [35]. Study demonstrated that noxious stimuli and non-noxious such as tactile stimuli and light pressure effectively elicited the improvement of WDR neurons response with the increase of stimulation intensity [35]. WDR neurons projecting to the contralateral spinal cord are the main neurons that modulates pain-related signaling in the spinal cord [56,57,58]. In this research, to further clarify the possible mechanisms by which how spinal BDNF leads to the pathogenesis of SNI-induced neuropathic pain, we observed the effect of BDNF on C-fiber responses of dorsal horn WDR neurons, which are greatly associated with nociceptive transmission [35]. We successfully recorded C-fiber-evoked discharges of WDR neurons in the spinal dorsal horn of SNI rats, and found that in comparison with sham rats, the C-fiber-evoked discharges of dorsal horn WDR neurons is obviously enhanced following SNI, implying that SNI can effectively improve the excitability of dorsal horn WDR neurons and induce central sensitization, which is in parallel to the other experiments [59]. The results also confirmed that the hyperexcitability of dorsal horn WDR neurons-mediated central sensitization may be one of the mechanisms for SNI-induced neuropathic pain. By contrast, 2 Hz EA treatment of SNI rats markedly reduced C-fiber-evoked discharges of WDR neurons in the spinal dorsal horn, indicating that the excitability of dorsal horn WDR neurons was also blocked by 2 Hz EA stimulation. To further explore the role of central sensitization in neuropathic pain development, after intrathecal injection of exogenous BDNF to the spinal dorsal horn of SNI rats, we presently found that the inhibitory effect of 2 Hz EA on the excitability of dorsal horn WDR neurons was significantly reversed by exogenous BDNF in SNI rats. Based on the evidence and our present data, from the view of the excitability of dorsal horn WDR neurons, the current experiment further verified that 2 Hz EA treatment relieved SNI-induced neuropathic pain by inhibiting the BDNF/TrκB signaling pathway-mediated hyperexcitability of dorsal horn WDR neurons, which is closely correlated with SNI-evoked central sensitization.

## 4. Materials and Methods

### 4.1. Experimental Animals

A total of 164 healthy male SD rats weighing 180–200 g were used in this experiment, which are provided by the Experimental Animal Center of Peking University Medical Department. Rats were housed five per cage with free access to food pellets and water, and they were kept in a temperature-controlled (22–24 °C) room with a 12/12 h light/dark cycle. All animal experimental protocols strictly followed the guidelines of International Pain Research Association and have been approved by the Animal Protection and Use Committee of Peking University in China, agreement number SYXK(J) 2016-0010, 23 November 2016. Every possible examination was conducted to minimize pain to rats.

### 4.2. Experimental Protocols

**Figure 8:**
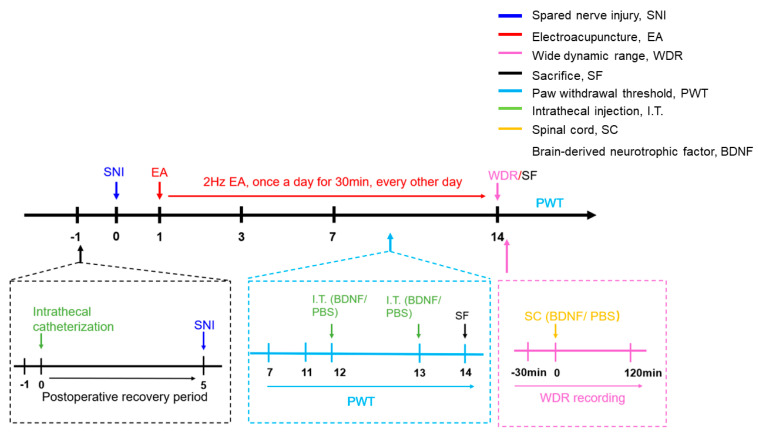
The schematic diagram of the experimental protocols.

Animal experimental procedures for SNI-induced neuropathic pain can be found in this research (Figure 8). For the first test, to determine the effect of 2 Hz EA on the mechanical hypersensitivity after SNI, rats were randomly assigned to one of 4 groups: sham, SNI, SNI + Mock EA, and SNI + EA groups. The sham rats underwent sham surgery, while the other groups received SNI surgery. Rats in the SNI + EA group were treated with 2 Hz EA once every other day on day 1 post-SNI surgery for 14 days. Rats in the sham, SNI, and SNI + Mock EA groups were received the same way as those in the SNI + EA group except for EA stimulation. In order to observe the effect of exogenous BDNF on 2 Hz EA producing the attenuation of mechanical hypersensitivity in SNI rats, intrathecally injected 100 ng BDNF or PBS on SNI rats starting from day 12 of 2 Hz EA treatment. The PWT was assessed following administration of 2 Hz EA on day 14 post-SNI surgery, respectively.

For the second test, to explore the potential mechanism underlying 2 Hz EA-mediated alleviation of neuropathic pain, rats were randomly divided into 6 groups: sham, SNI, SNI + Mock EA, SNI + EA, SNI + EA + BDNF, and SNI + EA + PBS groups, rats from each group were underwent to harvest the L4–L6 segments of spinal cord on day 14 post-surgery after behavioral experiments. The samples were collected for detecting the levels of BDNF/TrκB cascade in both mRNA and protein by immunofluorescence staining and Western blot assay.

For the third test, to further define the effect of C-fiber-evoked discharges of dorsal horn WDR neurons in 2 Hz EA-mediated alleviation of neuropathic pain, rats were randomly divided into 6 groups: sham, SNI, SNI + Mock EA, SNI + EA, SNI + EA + BDNF, and SNI + EA + PBS groups. The C-fiber-evoked discharges of dorsal horn WDR neurons was recorded to indicate the noxious response of WDR neurons by application of extracellular electrophysiological recording.

### 4.3. A Rat Model of Spared Nerve Injury (SNI)

After rats were adapted to the surrounding for 5 days, the rat model of spared nerve injury (SNI) was established according to the method described in previous study [28]. Briefly, rats were anesthetized with isoflurane (C002170901, YiPin Pharmaceutical, Shijiazhuang, Hebei, China) at a concentration of 2–3%. The left lateral sciatic nerve and its trifurcations (three terminal branches including sural, common peroneal and tibial nerves) were exposed. The left common peroneal and tibial nerves were tightly ligated with a 4-0 silk thread, being sectioned distal to the ligation with removal of 2–4 mm of the distal nerve stump, and keeping the sural nerve intact. The skin and muscle of rats were closed under sterile condition. Only rats that developed pain hypersensitivity were applied in this experiment. While the sham-operation rats were performed with all identical surgery protocols except that only exposing the tibial and peroneal, the common nerve was not ligated and cut.

### 4.4. Intrathecal Administration

After rats were anesthetized with 2–3% isoflurane, the implantation of intrathecal cannula to rats were performed as described previously study [28]. Briefly, a PE-10 polyethylene catheter (BB31695-PE/1, American Health & Medical Supply International Corp, New York, NY, USA) was implanted between the L5 and L6 vertebrae to the lumber enlargement level (3.5–4 cm rostral than the cannula) of the spinal cord. The outer part of the PE-10 catheter was plugged and fixed onto the skin between rat ears to close the wound. All surgery procedures were conducted under sterile condition. Two days post-surgery, only rats without the sign of spinal cord damage were used in this experiment. Before further experimental procedure was performed, the rats were given a 5 day recovery, and then the SNI-induced neuropathic pain rat model was established. According to the protocols described in previous report [9,27], in the experiment, after 2 Hz EA treatment of SNI rats on day 12, exogenous BDNF was intrathecally given for consecutive two days, twice a day at a dose of 100 ng. Intrathecal injection of BDNF or vehicle through the implanted catheter in a 20 μL volume of solution, then using 10 μL of normal saline (NS) to flush. The needle kept in situ for 2 min following each injection.

BDNF (GenScript, Nanjing, China) was first dissolved as a concentrated stock solution (0.5 μg/μL) in PBS and stored at −20 °C. Xing et al. [9] found that administration of exogenous BDNF at a dose of 100 ng can successfully induce mechanical hyperalgesia lasting for 7 days in rats. Thereby, in this research, the application of BDNF at an effective dose of 100 ng to further investigate the possible mechanisms underlying 2 Hz EA-mediated an inhibitory effect in SNI-induced neuropathic pain rats.

### 4.5. Assessment of Mechanical Hypersensitivity

Mechanical pain is a behavioral indicator for the detection of neuropathic pain, and the von Frey filaments (Stoelting, Wood Dale, IL, USA) were used to evaluate the paw withdrawal threshold (PWT) of rats using the “up and down” method as described by Berrocoso’ report [60]. Briefly, rats were placed in a plexiglass sleeve (18 × 6 × 6 cm) with a metal mesh at the bottom. After acclimating to the environment for 15 min, a series of standardized von Frey filaments were used according to 0.41, 0.70, and 1.20. The ascending order of 2.00, 3.63, 5.50, 8.50, and 15.10 g was applied to the plantar surface of the hind paw of the rat to observe whether there was a withdrawal reaction. Rats with rapid foot withdrawal reactions during the stimulation time or when the von Frey filaments were removed, which were recorded as positive reactions. If a positive reaction occurs, von Frey filament stimulation is given to the adjacent lower intensity, and this is continued until the first positive and negative (or negative and positive) reaction occurs, and then measured downward for consecutive 4 times. A few seconds are required between different stimulation to eliminate the effect of previous response. The 50% of PWT were calculated by this formulas as described previously [61]. Tests were performed on day 1 before surgery and on day 3, 7, 11, 12, 13, and 14 after surgery, respectively.

### 4.6. Electroacupuncture Stimulation

2 Hz EA treatment of SNI rats were conducted as described in our previous reports [23,28,29]. In brief, under without anesthesia states, rats were housed in specially designed fixtures with its both hind legs and tail exposed, the skin of hind legs in rat was sterilized using 75% alcohol. EA stimulation was performed on the bilateral acupoints of Zusanli (ST36, 5 mm lateral to the anterior tubercle of the tibia marked by a notch) and Sanyinjiao (SP6, 3 mm proximal to the medial malleolus and localized in the posterior border of the tibia) in rats. The square waves of EA stimulus were generated from Han’s Acupoint Nerve Stimulator (HANS, LH202, Huawei Industrial Developing Company, Beijing, China), which were used to stimulate the hind legs of rat simultaneously. EA stimulation started from day 1 post-SNI surgery and was conducted daily between 09:00 and 11:00. In this research, EA stimulation frequency was selected for 2 Hz with 0.6 ms pulse width, EA stimulation intensity was elevated in a gradient manner at 1-2-3 mA, and each stimulation intensity lasting for 10 min. 2 Hz EA treatment of rats with once every other day lasted for 14 days.

### 4.7. Quantitative RT-PCR Analysis

According to the method described in our previous report [29]. Briefly, after taking the material on the 14th day, the L4-L6 spinal cord tissue was placed into a 1.5 mL enzyme-free EP tube, and 10 times the volume of Trizol was added. The spinal cord tissue was lysed, and then 1/5 of chloroform was added, mixed upside down for 15–30 s, and left at room temperature for 5 min, and centrifuged at 12,000× *g* for 15 min at 4 °C. Then taken the upper aqueous phase, added equal volume of isopropanol, mixed well, left it at room temperature for 10 min, 4 °C, 12,000× *g*, and centrifuge for 10 min. DEPC water is equipped with 75% ethanol to wash the bottom of the sink. After being washed at 4 °C, 7500× *g*, centrifuged for 10 min. After centrifugation, the washed ethanol is discarded and dried. After the drying is completed, being quantified using about 20 μL of RNase Free in water. After reversing transcription was completed, a real-time quantitative PCR experiment was performed. qPCR reaction system: cDNA 2 μL, 1×SYBR 10 μL, add ultrapure water to 20 μL. Reaction conditions: pre-denaturation at 95 °C for 20 min, PCR reaction, denaturation at 95 °C for 10 s, annealing at 61 °C for 20 s, extension at 72 °C for 25 s, a total of 40 cycles. The primer sequences were synthesized by Shanghai Shenggong Biological Company, and the primer sequences are as the following (Table 1).

### 4.8. Western Blot Analysis

Following rats received deep anesthesia with 2% to 3% isoflurane, Western blot was performed as previously described [23,28,29]. In short, protein was extracted from L4–L6 segment of spinal cord tissue, 40 mg of protein per sample was separated using 8–10% SDS-PAGE, and microgels and transmembrane were used. Instrument (Bio-Rad, Hercules, CA, USA) was performed for Western blot analysis on PVDF (Millipore, Darmstadt, HD, Germany) membranes. Incubated the membrane with 5% non-fat milk in Tris buffered saline containing 0.1% Tween-20 for 1 h, and then used rabbit anti-BDNF antibody (1: 1000, ab108319, Abcam, Cambridge, UK) or p-TrκB (1: 1000, ab229908, Abcam, UK) or TrκB (1: 1000, 4603, CST) or GADPH (1: 1000, Solarbio, Beijing, China) and being incubated overnight. The blot was washed and incubated with horseradish peroxidase-conjugated anti-rabbit secondary antibody (1: 1000, Cell Signaling Technology, Danvers, MA, USA) for 1 h at room temperature. The site of the antigen-antibody complex can be seen with Immobilon Western chemiluminescence HRP substrate (Millipore, Darmstadt, CAHD, Germany). Bands were analyzed by Quantity One software (Bio-Rad). GADPH was used as an internal control. The values of BDNF, p-TrκB and TrκB were expressed as the ratio of the optical density of the band to the density of GADPH.

### 4.9. Extracellular Electrophysiological Recording

According to the method as described in previous research [35], after the SNI rats were anesthetized with urethane (1.2–1.5 g/kg), the right jugular vein and tracheal intubation were performed. T13 to L1 laminectomy was performed to expose most of the spinal lumbar enlargement. During the operation, the heart rate of the rats was maintained at 330–460 beats/min, the end-expiratory carbon dioxide concentration was 3.5–4.5%, and the rectal temperature was 36.5–37.5 °C. Observed the above monitoring indicators of the rats at any time, and terminated this experiment, if it exceeded the normal range. A para-xylene-coated tungsten microelectrode (impedance 4 MΩ, FHC Inc., Bowdoinham, ME, USA) was placed on the spinal cord lumbar enlargement for extracellular wide-dynamic range (WDR) neurons recording. The stepping microelectrode thruster pierced the tungsten wire microelectrodes vertically in a 1 μm stepping method. During the microelectrode advancement, a test square wave electric pulse stimulation of 1/2 s was given to find neurons without spontaneous discharge. The neurons that respond to non-noxious stimuli such as skin contact and light pressure, and toothed forceps, as well as the intensity of the response increases with the increase of the stimulus intensity are clearly WDR neurons for further study.

During the experimental recording, rats were firstly given 3 experimental sciatic nerves each with 10 square wave stimuli (frequency 0.5 Hz, wave width 0.5 ms), and intensity 2 times the C-fiber response threshold (0.5–5.0 mA). The experimental electrical stimulation, and its average value as the basic control value (100%). BDNF at the dose of 100 ng is then administered on the surface of spinal cord. Repeated string stimulation was performed every 5 min. The changes in evoked-discharges of WDR neurons within 120 min after administration of BDNF (with PBS as a control) were recorded. The recorded unit discharge was input into the memory oscilloscope through the microelectrode amplifier, while it was input into the Spike2 bioelectric signal acquisition and analysis system (CED, UK). A series of WDR neurons discharges were superimposed to draw a histogram of the sequence density after stimulation, and the further data processing and statistical analysis were performed.

### 4.10. Statistical Analysis

In this experiment, the Prism 8.0 software was used for data processing and statistical analysis. Difference between two groups was compared with *t*-tests. One-way analysis of variance (ANOVA) followed by Newman–Keuls post hoc for multiple comparison test or two-way ANOVA followed by Bonferroni post-hoc test was used for multiple comparison. All experimental data were expressed as the mean ± SEM. *p* < 0.05 was considered statistically significance.

## 5. Conclusions

**Figure 9:**
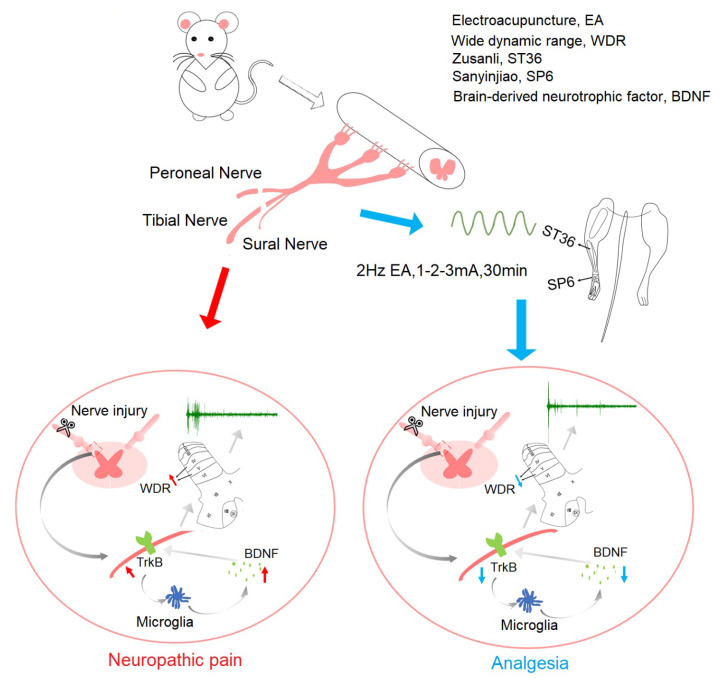
Schematic diagram illustrating the potential mechanisms underlying 2 Hz EA-mediated analgesic effect on SNI-induced neuropathic pain. Administration of 2 Hz EA to SNI rats alleviated mechanical hypersensitivity via the inhibition of BDNF/TrκB signaling cascade-mediated hyperexcitability of dorsal horn WDR neurons in the spinal cord.

In summary, we currently found that 2 Hz EA exerted an inhibitory effect on mechanical hypersensitivity, down-regulated the spinal levels of BDNF and TrκB in both mRNA and protein, and suppressed the excitability of dorsal horn WDR neurons in SNI rats, while intrathecally injected BDNF effectively reversed this inhibitory effect of 2 Hz EA on SNI rats (Figure 9). This experiment confirmed that the analgesic effect of 2 Hz EA on SNI rats is likely to be related to blockade of spinal BDNF/TrκB signaling cascade-mediated central sensitization, and indicated that blockade of BDNF/TrκB maybe a promising approach for 2 Hz EA ameliorating neuropathic pain.

## Figures and Tables

**Table 1:** The PCR primer sequences.

Genes	Sequences
*GADPH-F*	CAGCCGCATCTTCTTGTGC
*BDNF-F*	GGTAACCAGGCGTCCGATA
*BDNF-R*	CCAGATCCTCCCTGACTGGT
*TrκB-F*	TAAGGCTGAATGGTGTGCGT
*TrκB-R*	CTGCCTTCACAGATACCCAGTA
-	ACCTTAAACTCGGACCTCACC
